# External quality assessment to support the WHO ProSPeRo study for the evaluation of two dual HIV/syphilis point-of-care tests in seven countries

**DOI:** 10.1186/s12879-024-09027-3

**Published:** 2024-02-29

**Authors:** Weiping Cao, Yetunde F. Fakile, Mayur R. Shukla, Kevin Pettus, Kathryn Lupoli, Jaeyoung Hong, Allan Pillay, Ranmini Kularatne, Hicham Oumzil, Valeska Padovese, Nigel Sherriff, Isaac SSewanyana, Silver K. Vargas, Antonella Zorzi, Karel Blondeel, Igor Toskin, Ellen N. Kersh

**Affiliations:** 1 Division of STD Prevention, Centers for Disease Control and Prevention, Atlanta, GA 30329 USA; 2 Division of Global HIV &TB, Centers for Disease Control and Prevention, Atlanta, GA USA; 3 Centre for HIV & STI, National Institute for Communicable Diseases, Johannesburg, South Africa; 4 National Reference Laboratory for HIV, Virology Department, National Institute of Hygiene, and Pedagogy and Research Unit of Microbiology, School of Medicine and Pharmacy, Mohammed V University in Rabat, Rabat, Morocco; 5 Genitourinary Clinic, Department of Dermatology and Venereology, Mater Dei Hospital, Msida, 2090 Malta; 6 School of Sport and Health Sciences, University of Brighton, Brighton, UK; 7 Central Public Health Laboratories, Ministry of Health, Plot 1062, 106 Old Butabika Rd, Kampala, Uganda; 8 Center for Interdisciplinary Research in Sexuality, AIDS and Society, Universidad Peruana Cayetano Heredia, Lima, Peru; 9 Virology and Microbiology Unit, Department of Pathology and Diagnostics, Verona University Hospital, Verona, Italy; 10 Virology and Microbiology Unit, Department of Molecular Medicine, Padua University Hospital, Padua, Italy; 11 Department of Sexual and Reproductive Health and Research, UNDP-UNFPA-UNICEF-WHO-World Bank Special Programme of Research, Development and Research Training in Human Reproduction (HRP), World Health Organization, Geneva, Switzerland; 12 Faculty of Medicine and Health Sciences, Ghent University, Ghent, Belgium

**Keywords:** External Quality Assurance (EQA), Dried tube specimens (DTS), Point-of-care diagnostic tests (POCTs), Dual HIV/syphilis assay, Sexually transmitted infections (STIs)

## Abstract

**Background:**

Sexually transmitted infections (STIs) such as syphilis and HIV remain to be a significant public health issue worldwide. Dual rapid point-of-care tests (POCTs) have shown promise for detecting antibodies to HIV and syphilis but have not been fully evaluated in the field. Our study supported the WHO ProSPeRo study on Sexually Transmitted Infection Point-of-Care Testing (STI POCT) by providing external quality assessment (EQA) for HIV and syphilis testing in reference laboratories and their associated clinical sites in seven countries.

**Methods:**

HIV/syphilis serum liquid and dried tube specimen (DTS) panels were prepared by CDC. Liquid panels were distributed to the reference laboratories for three rounds of testing using commercially and locally available laboratory-based serological tests. DTS panels were sent to the clinical testing sites for 8 rounds of POC testing using the Abbott SD BIOLINE HIV/Syphilis Duo test (hereafter referred to as SD BIOLINE) and the Chembio Dual Path Platform (DPP) HIV-Syphilis assay. EQA panels were tested at CDC using the Rapid Plasma Reagin (RPR) test and the *Treponema pallidum* Particle Agglutination assay (TP-PA) for syphilis antibodies. Genetic Systems HIV-1/HIV-2 Plus O EIA, Geenius HIV Supplemental Assay and the Oraquick Advance HIV test were used to detect HIV antibodies in the EQA panels. Results from the reference laboratories and POCT sites were compared to those obtained at the CDC and a percentage agreement was calculated.

**Results:**

Qualitative RPR and TP-PA performed at the reference laboratories demonstrated 95.4–100% agreement with CDC results while quantitative RPR and TP-PA tests demonstrated 87.7% and 89.2% agreement, respectively. A 93.8% concordance rate was observed for qualitative HIV testing in laboratories. EQA testing at clinical sites using dual tests showed 98.7% and 99.1% agreement for detection of HIV antibodies and eight out of 10 sites had > 95.8% agreement for syphilis testing. However, two clinical sites showed only 65.0–66.7% agreement for SD BIOLINE and 84.0–86.7% for DPP, respectively, for syphilis testing.

**Conclusions:**

Overall, laboratories demonstrated high EQA performance in this study. Both HIV/syphilis POCTs gave expected results in the clinic-based evaluations using DTS. However, testing errors were identified in a few testing sites suggesting the necessity for continuous training and monitoring the quality of POC testing.

## Background

Sexually transmitted infections (STIs) continue to be a major public health issue worldwide, particularly in under-resourced settings. More than 1 million new STIs cases are acquired every day, the majority of which are asymptomatic [1]. Syphilis is an infection caused by the bacterium *Treponema pallidum* subspecies pallidum (*T. pallidum*). In 2020, the World Health Organization (WHO) estimated that 7.1 million new syphilis infections occurred worldwide [2]. The human immunodeficiency virus (HIV) attacks the body’s immune system and, if left untreated, it can lead to acquired immunodeficiency syndrome (AIDS). At the end of 2021, an estimated 38.4 million people worldwide are thought to be living with HIV and an estimated 1.5 million people recently acquired HIV infection [3]. Both syphilis and HIV can be passed from mother to child. Approximately one million pregnant women (PW) have active syphilis globally each year, resulting in over 600,000 cases of congenital syphilis and greater than 350,000 adverse birth outcomes [4]. In 2015, more than 1.4 million pregnant women were infected with HIV, and mother-to-child transmission (MTCT) of HIV is estimated to have resulted in over 150,000 infant cases [5]. In 2014, WHO called for elimination of mother-to-child transmission (EMTCT) of HIV and syphilis [6].

HIV and syphilis co-infection is evident in the United States and many other developed societies with robust surveillance systems. Both epidemics predominantly affect men who have sex with men (MSM) and other at-risk populations, such as sex workers and persons who use drugs [7, 8]. Untreated HIV infection may modulate the clinical presentation of syphilis and increase the risk for neurologic complications and treatment failure [9]. Additionally, syphilis facilitates transmission and acquisition of HIV [10] and has a transient negative impact on the course of HIV infection with increased viral load and a decrease in the CD4 cell count [11, 12]. Concurrent syphilis infection in pregnant women living with HIV is significantly associated with vertical perinatal HIV transmission [13]. The substantially higher economic burden and lower survival rate associated with late entry into medical care make early diagnosis essential [14]. Therefore, rapid, accurate and easy-to-use dual HIV/syphilis tests provide simultaneous screening for the two diseases and could therefore substantially benefit patients and reduce the economic burden to health services. This is especially critical for pregnant women and key populations including men who have sex with men (MSM), sex workers and persons who use drugs.

Serological testing has long been the primary tool for diagnosing both syphilis and HIV infection. In the laboratory, both HIV antigens and antibodies can be identified using serological testing [15]. A diagnosis of syphilis requires the use of both treponemal tests (detection of antibody to *T. pallidum-*specific proteins) and nontreponemal tests (detection of antibodies against lipoidal antigens from damaged host cells and treponemes) to confirm active or past/treated syphilis infection.

Introduction of single, disease-specific point-of-care tests (POCTs) has been instrumental in scaling-up both HIV and syphilis testing, especially in resource limited settings. In 2019, the rapid dual HIV/syphilis test was recommended by WHO as the first-line test for pregnant women attending antenatal care [16]. The Abbott SD BIOLINE HIV/Syphilis Duo test (Abbott Point of Care Diagnostics, Princeton, NJ, hereafter referred to as SD BIOLINE) and the Chembio Dual Path Platform (DPP) HIV-Syphilis assay (Chembio Diagnostics, Medford, NY, hereafter referred to as Chembio DPP) are both single-use rapid, qualitative, multiplex lateral-flow immunoassays for the detection of antibodies to HIV-1/2 and *T. pallidum* simultaneously. Chemio DPP was the first dual rapid test approved by FDA in 2020 and also received Clinical Laboratory Improvement Amendments (CLIA) waiver from FDA in 2023. SD Bioline HIV/Syphilis Duo test has been awarded WHO prequalification, making it the first dual HIV/syphilis point-of-care test available for deployment in national screening programs targeting resource-limited countries. Accurate results of rapid testing at point-of-care have a direct impact on establishment of a diagnosis, provision of treatment and prevention of further disease spread. However, there are limited data on the performance of dual HIV/syphilis tests in field settings [17, 18]. The global ProSPeRo study (Project on Sexually Transmitted Infection Point-of-care Testing) was established to address the need for standardized high-quality evaluation of STI POCTs which is critical for the further development and global uptake [19, 20]. This study, an integral part of ProSPeRo, was designed to assess the need for external quality assessment (EQA) for HIV and syphilis serological testing in both reference laboratories and their associated clinical sites that were participating in a stringent evaluation of dual HIV/syphilis POCTs in seven countries.

## Methods

### Participating laboratories and clinical sites

The dual HIV/syphilis EQA program was implemented in seven countries between 2018 and 2020. Reference laboratories that were not already taking part in an international EQA program and all ProSPeRo study sites were invited to participate in this study. Participating laboratories included the Laboratory of Sexual Health, Universidad Peruana Cayetano Heredia, Lima, Peru; Microbiology and Virology Unit, Verona University Hospital, Verona, Italy; Virology, Bacteriology and Molecular Diagnostics, Mater Dei Hospital Laboratory, Msida, Malta; Microbiology and Infection Laboratory, Royal Sussex County Hospital, Brighton, United Kingdom; HIV and STIs Reference Laboratories, National Institute of Hygiene, Rabat, Morocco; Centre for HIV & STI, National Institute for Communicable Diseases, Johannesburg, South Africa and Uganda National Health Laboratory, Kampala, Uganda. On-site EQA and technical training was provided by WHO to staff of all participating sites before commencement of the study.

### Preparation of serum liquid and DTS panels

EQA liquid and dried tube specimen (DTS) panels were prepared at the STD Laboratory Reference and Research Branch (STDLRRB) of the US Centers for Disease Control and Prevention (CDC) following published methods [21, 22]. Briefly, HIV or syphilis reactive and non-reactive serum samples with volumes ranging from 200 to 700 ml were purchased from commercial sources (Plasma Service Group, PA, USA and Physician’s Plasma Alliance, TN, USA) and tested at CDC to confirm the reactivity for HIV and syphilis as described below. Each EQA liquid and DTS panel consisted of 5 serum specimens: one syphilis reactive only, one HIV reactive only, one reactive for both syphilis and HIV, one non-reactive for both syphilis and HIV and one biologically false positive (BFP) syphilis serum. A BFP was defined as a serum sample that was reactive with a nontreponemal test but nonreactive with treponemal tests [23]. To prepare reactive sera for both syphilis and HIV, syphilis and HIV reactive specimens were mixed in a 1:1 ratio and samples were tested to confirm expected results at CDC.

For the preparation of liquid EQA panels [22], 1.5 ml of each sample were dispensed into sterile glass vials under a laminar flow hood. Vials were then sealed, labelled, and stored refrigerated until shipped. For the preparation of DTS panels [21], each serum specimen was mixed with 0.1% (v/v) of green food colouring dye (McCormick, Hunt Valley, MD), 20 μL of the solution was dispensed into 2 ml plastic vials and left open in a laminar flow hood overnight for drying. Vials were then capped, labelled, and stored refrigerated until shipped.

### Quality control (QC) and reproducibility testing of EQA panels at CDC

Five serum specimens included in the liquid and DTS EQA panels were tested at CDC using the qualitative and quantitative Rapid Plasma Reagin test (RPR, Arlington Scientific, Springville, UT) to detect nontreponemal antibodies and the *T. pallidum* Particle Agglutination assay (Serodia TP-PA, Fujirebio, Malvern, PA) to detect *T. pallidum* specific treponemal antibodies, following the manufacturers’ instructions. EQA panels were also tested using Genetic systems HIV-1/HIV-2 Plus O EIA (Bio-Rad Laboratories, Hercules, CA), Geenius HIV Supplemental Assay (Bio-Rad Laboratories) and Oraquick Advance HIV test (OraSure Technologies, Bethlehem, PA) to detect HIV antibodies. To assess the reproducibility of rapid tests on DTS EQA panels, the panels were reconstituted and tested (described below) using two FDA-cleared syphilis single or dual rapid tests, Syphilis Health Check (SHC, Diagnostics Direct, Stone Harbor, NJ) and Chembio DPP, following the manufacturers’ instructions for five consecutive days by the same tester.

### Testing of liquid/DTS panels at laboratories and POCT sites

Each liquid/DTS EQA panel contained 5 vials of sample labelled A1 to A5, instructions for handling and testing and a result recording form. Each DTS panel also included one vial of reconstitution PT buffer containing 0.1 M Phosphate Buffered Saline (PBS) with 0.1% Tween-20 (pH 7.4, Sigma) and two plastic pipettes. Liquid panels were tested by laboratories using commercially and locally available laboratory-based serological tests. Both TP-PA/TP-HA (Treponema pallidum Haemagglutination) and RPR tests were qualitatively and quantitatively reported. DTS panels were tested at associated clinical sites using both SD BIOLINE and DPP dual HIV/syphilis tests. The day before testing, each DTS vial was reconstituted by adding 4 drops (200 µl) of PT buffer, mixed well and incubated overnight at room temperature to allow solubilization of dried serum into the PT buffer. On the test day, DTS panel sample tubes were mixed gently by tapping and subsequently tested following the manufacturers’ instructions for serum/plasma specimen type.

### Implementation of EQA

**Fig. 1:**
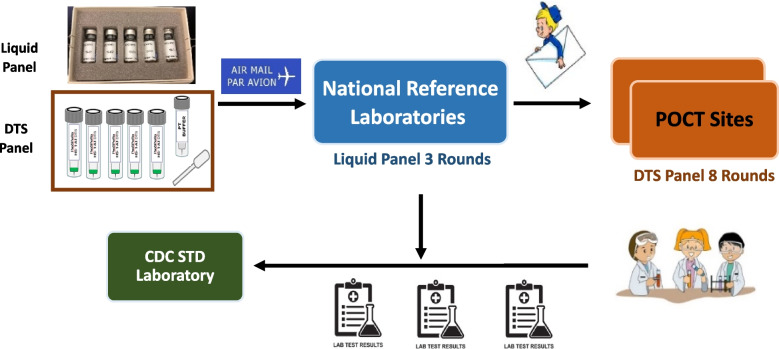
Schematic of EQA implementation workflow. Liquid and DTS EQA panels were prepared at CDC STDLRRB. All panels were shipped to reference laboratories who then distributed DTS panels to clinical POCT sites. All test results were sent back to CDC STDLRRB for grading

Both liquid and DTS EQA panels were shipped from -CDC to the laboratories in each participating country and DTS panels were then distributed by reference laboratories to the clinical POCT sites (Fig. 1). The laboratories conducted three rounds of EQA testing using liquid panels over a one-year period and 2–3 testers at each of the clinical POCT sites performed eight rounds of EQA testing using DTS panels over a 16-month period. All EQA panels were color coded and labelled appropriately to reflect the round number. Five serum specimens in each EQA panel were randomized for each round of testing and all sites received the same panel for each round. All test results from laboratories and POCT sites were returned to CDC for grading.

### Grading of results

#### Grading reference laboratory test results

Laboratory test results for detection of HIV and *T. pallidum* antibodies were compared to results obtained at CDC STDLRRB. Each round of testing was assigned a maximum score of 100 points for 5 specimens (20 points for each specimen). The passing score for each round was 80. The overall grade was an average of all the tests performed by each laboratory. For HIV and syphilis qualitative tests, an incorrect result was scored 0. For quantitative RPR and TP-PA tests pertaining to reference labs, results were expressed as a titer (the reciprocal of the highest dilution at which the tested sample produced a reactive result). A quantitative result was scored 20 points if titers were within one dilution (two-fold) above or below expected results; scored 10 points if outside one dilution but within two dilutions (fourfold); scored 0 points if the result was outside two dilutions of the expected value.

#### Grading POCT site test results

Each DTS panel containing 5 samples was tested by 1–3 testers at each POCT site. Each dual test (SD BIOLINE and DPP) generated results for both HIV and syphilis. Therefore, there were four test results for each sample in the DTS panel: Chembio syphilis, Chembio HIV, SD Bioline syphilis and SD Bioline HIV. Each correct result was assigned 5 points, thus readings for each specimen could yield maximum of 20 points. Thus, each round of 5 specimens could score a maximum of 100 points. The score of each round was taken as the average score of all testers. The score for each site was the average score of all test rounds conducted at that site.

### Statistical analysis

All results were analyzed using SPSS (IBM®SPSS for Windows, Version 21.0) and R software [24]. Results from laboratories and clinical POCT sites were compared to those obtained by CDC, which served as the reference standard. The percentage agreement was calculated as following:

% agreement for reference labs = (total sample number—qualitative incorrect or titer incorrect sample number)/total sample number × 100% agreement for POCT sites = (total sample number -false positive-false negative-indeterminate sample number)/total sample number × 100 The Cohen’s kappa coefficient (κ) was used to measure the agreement between testing at CDC STDLRRB and reference labs or POCT sites.

## Results

### QC results of samples included in the EQA panel

The QC results obtained with 5 serum samples (A1-A5) included in the EQA panel were summarized in Table 1. Sample A1 was HIV reactive, but non-reactive for both treponemal and nontreponemal antibodies. Sample A2 was HIV non-reactive but reactive for treponemal antibodies. The RPR was also reactive for sample A2 with a titer of 1:2. Sample A3 was a negative control and had no detectable antibodies to both HIV and syphilis. Sample A4 was reactive for both HIV and syphilis with an RPR titer of 1:1. Sample A5 was a biological false positive serum showing non-reactivity to HIV and treponemal antibody testing but was reactive by RPR testing (titer 1:4). The reproducibility study of EQA DTS panel demonstrated consistent results during 5 consecutive days of testing at CDC STDLRRB using SHC and Chembio tests (data not shown). There were a total 592 to 607 vials of DTS produced for each sample at CDC for this study (Table 1).

**Table 1:** QC results of samples included in the EQA panel

Sample ID	TP-PA titer	RPR titer	Genetic System	Geenius	Oraquick	Result	DTS vial produced
A1	NR	NR	R	R	R	HIV R	592
A2	1:20480	1:2	NR	NR	NR	SYP R	598
A3	NR	NR	NR	NR	NR	HIV/SYP NR	606
A4	1:10240	1:1	R	R	R	HIV/SYP R	607
A5	NR	1:4	NR	NR	NR	BFP	599

*RPR* Rapid Plasma Reagin, *TP-PA T.pallidum* particle agglutination assay, *Genetic System* Genetic systems HIV-1/HIV-2 Plus O EIA, *Geenius* Geenius HIV Supplemental Assay, *Oraquick* Oraquick Advance HIV test, *DTS* Dried tube specimen, *SYP* Syphilis, *R* Reactive, *NR* Non-reactive, *BFP* Biologically false positive

### Participating laboratories and POCT sites

From 2018 to 2020, laboratories (Labs 1–7) in seven countries including Morocco, South Africa, Uganda, Italy, Malta, United Kingdom (UK) and Peru from three continents (Africa, Europe and South America) participated in this study. Four out of seven laboratories completed all three rounds of testing. One laboratory (Lab1) was only able to complete one round while two laboratories (Lab6 and Lab7) did not perform any rounds of EQA to date as they already participated in international EQA schemes (Table 2). Overall, 15 rounds of testing (3 rounds per laboratory for Lab 1–5) were expected from all laboratories, of which 13 rounds were performed with a participation rate of 86.7%. The number of clinical POCT sites recruited in each country varied from 1 to 4. Eight rounds of testing with 3 testers per site were expected from each POCT site; however, the actual number of EQA rounds in each site as well as the number of testers for each round varied from 0 to 8 across the EQA program (Table 1). Ten out of 14 sites (71.4%) performed at least one round of testing. A total of 50 rounds of testing were performed on all sites. A total of 140 reports from 14 POCT testing sites were submitted for review.

**Table 2:** Participating laboratories and POCT sites

	**Laboratory**	**Rounds tested in labs**	**Number of POCT sites**	**Rounds tested in sites**	**Number of testers for each round per sites**
	**Lab1**	1	4	0	0
	**Lab2**	3	2	16	3
	**Lab3**	3	1	4	3
	**Lab4**	3	3	11	1–3
	**Lab5**	3	1	6	3
	**Lab6**	NA	2	8	2–3
	**Lab7**	NA	1	5	3
**Total**	**7**		**14**	**50**	

### EQA performance of laboratories

**Fig. 2:**
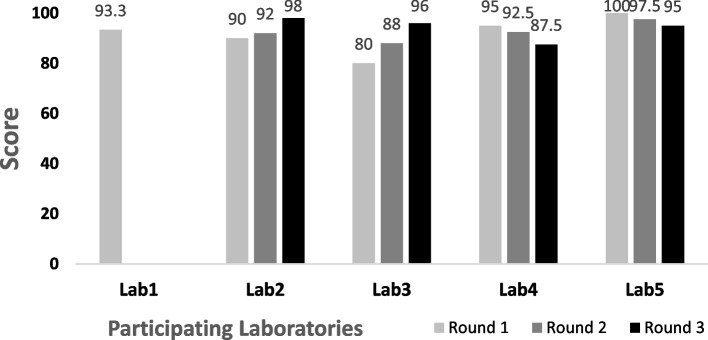
EQA scores for participating laboratories. EQA liquid panels (5 serum samples per panel) were tested at each participating laboratory (Lab1-Lab5) using the actual test kits and reagents that they routinely use in the laboratory. The overall score for each laboratory was an average of all the tests performed. Score for each round from each participating laboratory was listed in a bar chart

Five laboratories (Lab1-5) participated in the EQA program (Table 2). Four laboratories completed three rounds of testing. Lab1 performed round 1 testing and did not participate in EQA round 2 and 3. A total of 13 rounds of testing was completed by laboratories with 5 specimens for each round and a total of 65 specimens in total. As shown in Fig. 2, all laboratories scored above 80 for each test round which resulting in a 100.0% pass rate. The average scores for Lab1 to Lab5 were 93.3, 93.3, 88.0, 91.7 and 97.5, respectively. As shown in Table 3, for syphilis testing, 3 samples had discordant results for qualitative nontreponemal RPR tests when compared to the results from CDC. The overall agreement for qualitative RPR testing was 95.4% with a kappa value of 0.91, which suggests very good agreement. For RPR quantitative testing, 8 discordant results were observed in the laboratories, and all had titers ≥ fourfold higher as compared to reference results. The overall agreements were 87.7% and kappa value was 0.76, suggesting moderate agreement. Results for qualitative treponemal TP-PA/TP-HA testing from the laboratories had 100.0% agreement with reference results. There were 11 discordant results identified in all rounds of testing from laboratories for quantitative TP-PA/TP-HA testing and all had titers outside 2-dilutions compared to reference results, which resulted in 82.5% agreement and 0.46 kappa value. Lab5 did not determine the actual TPHA endpoint titer. For qualitative HIV testing, 4 samples gave discordant results demonstrating a 93.8% concordance rate and a kappa value of 0.90.

**Table 3:** EQA performance for each test type in laboratories

**Laboratory**	**Total Sample tested**	**RPR**		**TP-PA/TP-HA**		**HIV**
		Qualitative Discordant	**Quantitative Discordant**	Qualitative Discordant	**Quantitative Discordant**^**a**^	Qualitative Discordant
Lab1	5	1	0	0	1	0
Lab2	15	0	5	0	0	1
Lab3	15	1	3	0	5	3
Lab4	15	1	0	0	5	0
Lab5	15	0	0	0	3	0
**Total**	**65**	**3**	**8**	**0**	**14**	**4**
**Agreement**		**95.4%**	**87.7%**	**100.0%**	**82.5%**	**93.8%**
**Kappa**		**0.91**	**0.76**	**1.00**	**0.46**	**0.90**

^**a**^Specimen samples that did not have actual end-point titers or on which no testing occurred by lab were excluded from the % agreement and Kappa calculations

### EQA performance of clinical POCT sites

**Fig. 3:**
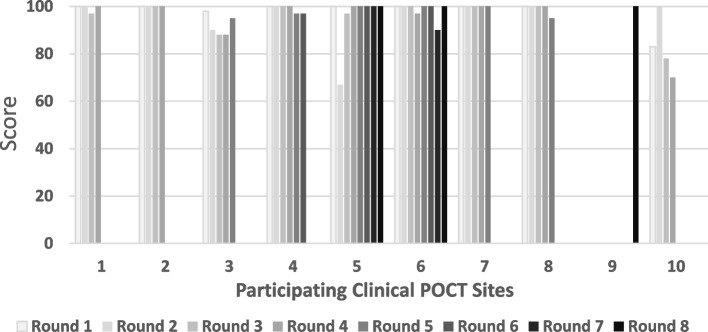
EQA score for each test round in clinical POCT sites. EQA DTS panels (5 serum specimens) were tested in each clinical POCT site using both Abbott SD BIOLINE HIV/syphilis Duo test and Chembio DPP HIV-Syphilis assay. Results from the POCT sites were graded using results from CDC as the reference method. An average score from all testers for each round in each site was listed in a bar chart

Among 140 sample test results submitted from the sites, 132 results had a passing score (> 80) with a pass rate of 94.3%. The average score among all 10 sites ranged from 82.8 to 100.0 with an average of 96.2 (Fig. 3). There were 36 reports with one or more discordant results as compared to the reference data from CDC. The performance of each dual rapid test for detection of *T. pallidum* antibodies was listed by site in Table 4. Overall, POCT sites achieved 94.4 and 94.1% agreement of DPP and SD BIOLINE test results, respectively. The overall kappa values were 0.775 and 0.782 for DPP and SD BIOLINE tests, respectively, suggesting a moderate level of agreement with the reference results. Noticeably, 8 out of 10 POCT sites had percent agreements above 95.8% (Table 4). At site 3, there were 10 and 12 false negative syphilis test results using DPP or SD BIOLINE rapid test, respectively. This resulted in 86.7% agreement (for DPP) and 84.0% (for SD BIOLINE) between site 3 and CDC. There were 19 false negative and 1 indeterminate result using the DPP test for detection of *T. pallidum* antibodies at site 10, while 18 false negative and 3 indeterminate results were observed using the SD BIOLINE test for syphilis antibody detection. These led to 66.7% agreement for DPP and 65.0% for SD BIOLINE for syphilis testing between site 10 and CDC. For the detection of HIV antibodies in all POCT sites, most test results from DPP and SD BIOLINE were consistent with reference results with overall 98.7%/ 99.1% agreement and a 0.958 / 0.979 kappa value, respectively (Table 5).

**Table 4:** Performance of dual HIV/syphilis test for detection of *T. pallidum* antibodies at POCT sites

Site	**Chembio DPP**					**Abbott SD BIOLINE**				**Agreement**
	**False + **	**False -**	**Indeterminate**	**Total**	**Agreement**	**False + **	**False -**	**Indeterminate**	**Total**	
1	0	0	1	55	98.2	0	0	0	55	100.0
2	0	0	0	60	100.0	0	0	0	60	100.0
3	0	10	0	75	86.7	0	12	0	75	84.0
4	0	0	0	90	100.0	0	0	2	90	97.8
5	1	2	0	120	97.5	1	2	0	120	97.5
6	0	5	0	120	95.8	0	3	0	120	97.5
7	0	0	0	50	100.0	0	0	0	50	100.0
8	0	0	0	60	100.0	0	0	0	60	100.0
9	0	0	0	10	100.0	0	0	0	10	100.0
10	0	19	1	60	66.7	0	18	3	60	65.0
Total	**1**	**36**	**2**	**700**	**94.4**	**1**	**35**	**5**	**700**	**94.1**
Kappa					**0.78**					**0.78**

**Table 5:** Performance of dual HIV/syphilis test for detection of HIV antibodies at POCT sites

Site	**Chembio DPP**					**Abbott SD BIOLINE**				**Agreement**
	**False + **	**False -**	**Indeterminate**	**Total**	**Agreement**	**False + **	**False -**	**Indeterminate**	**Total**	
1	0	0	1	55	98.2	0	0	0	55	100.0
2	0	0	0	60	100.0	0	0	0	60	100.0
3	0	0	0	75	100.0	0	0	0	75	100.0
4	0	0	0	90	100.0	0	0	2	90	97.8
5	1	3	0	120	96.7	1	3	0	120	96.7
6	0	0	0	120	100.0	0	0	0	120	100.0
7	0	3	0	50	94.0	0	0	0	50	100.0
8	0	0	0	60	100.0	0	0	0	60	100.0
9	0	0	0	10	100.0	0	0	0	10	100.0
10	0	1	0	60	98.3	0	0	0	60	100.0
Total	**1**	**7**	**1**	**700**	**98.7**	**1**	**3**	**2**	**700**	**99.1**
Kappa					**0.96**					**0.98**

## Discussion

With the high global burden of HIV and syphilis, especially in pregnant women and key populations, introduction of quality POC testing is extremely important to bring the diagnostic testing close to the site where clinical care is delivered. Dual HIV/syphilis rapid testing has the additional advantage of detecting two infections simultaneously using the same specimen. WHO has recommended using dual HIV/syphilis rapid tests for pregnant women since 2019, not only as the first test in antenatal care to help countries achieve EMTCT for both HIV and syphilis, but also as an important cost-saving measure [25]. New reduced costs of dual HIV/syphilis POCTs were recently announced to accelerate EMTCT progress [26]. There are many challenges associated with POCT to ensure test quality. Successful participation in an established EQA program should provide objective evidence of the testing sites’ competence for POC testing of specimens not only in laboratories, but also in clinical field POCT sites. If any technical and methodological problems are identified in EQA, it could help participating sites to improve the quality of testing. EQA for POCT sites could be organized and overseen by the local or national laboratories, who in their turn should prove their competence by participating in (inter)national EQA programs.

Overall, the reference laboratories that participated in the evaluation demonstrated good EQA performance and all labs passed the EQA syphilis and HIV testing. Nontreponemal test titers are useful for monitoring efficacy of treatment progression and disease status (past/treated, relapse or active infection). Patients successfully treated for syphilis typically show a fourfold (two doubling dilutions) decline in nontreponemal antibody titer over time, while a fourfold increase indicates either treatment failure or reinfection [27]. Lab1 and Lab2 had 3 and 5 discordant RPR results respectively, with all discordant RPR results having titers ≥ fourfold dilutions above expected results. Overall, TP-PA/TP-HA quantitative tests demonstrated 82.5% agreement with those of the CDC laboratory. There were 1 discordant TP-PA/TP-HA results for Lab1 and 5 discordant results for Lab3 and Lab4. All these TP-PA/TP-HA discordant results had titers outside 2 dilutions of reference results. Variability in nontreponemal and treponemal quantitative test results between different laboratories may have been due to test kits from different manufacturers being used and this has been reported in earlier studies [28, 29]. Therefore, it is recommended to use the same syphilis serologic test, preferably from the same manufacturer and same laboratory to monitor treatment response [27]. In addition, different laboratory personnel reading the test may have accounted for the discrepant results. The findings in this study indicate that any technically competent reference laboratory which undertakes evaluations of new POC tests for HIV and/or syphilis, or supervises the initiation of a program to use POC tests for these diseases at remote sites be, themselves, subject to a national or international EQA program. Including samples with low RPR titers in the EQA panel was extra challenging. Our study showed that only one laboratory missed one specimen with a low-reactive RPR (titer 1:4). All other laboratories successfully detected specimens with low RPR titers (1:1–1:4).

Dried tube specimens (DTS) were previously demonstrated to be a simple and cost-effective method for preparing quality assurance materials for use in resource-limited settings [21] and this method was adopted in our EQA program for the POCT sites. DTS were successfully distributed by reference laboratories to remote clinical sites, where the POC testing was ultimately performed by non-laboratory personnel. After appropriate training, the staff at the clinical sites were able to reconstitute DTS panels and perform the rapid tests following the manufacturers’ instructions. Overall, sites performed well in the EQA program using both the Abbott SD BIOLINE and Chembio DPP HIV/syphilis rapid tests. For the detection of *T. pallidum* antibody, results from DPP and SD BIOLINE dual tests demonstrated average 94.4% and 94.1% agreement with results from CDC. For the detection of HIV antibodies, 98.7–99.1% result agreement was observed in this study for both tests with Kappa values 0.96–0.98. Percentage of agreement was used to evaluate each lab- or site-specific testing performance and kappa value was calculated for the overall difference between lab/site and reference lab. For qualitative HIV testing, POCT sites demonstrated higher agreement than the reference laboratories. In a published laboratory evaluation study, the sensitivities for HIV detection were 98.2–100% for both SD BIOLINE and DPP HIV/syphilis test respectively, while for the detection of treponemal antibodies, the sensitivities were 86.5% and 85.0%, respectively [30]. Lower syphilis sensitivities of 66.2% and 68.6% were reported respectively for SD BIOLINE and DPP in a field performance evaluation study [18]. In our study, high false negative rates (15–31%) were observed for syphilis testing for both SD BIOLINE and DPP tests at site 3 and 10, which are located in the continent of Europe and Africa respectively (Table 3). However, these DTS panels performed well in eight other testing sites and results from the same sample were correctly detected for HIV antibodies on the same dual test device. These noted discrepancies seem to be site specific and not related to a specific tester at those two sites since errors mostly occurred in the same specimens among all testers. Differentiation between weak positive and negative syphilis test reactions could be a challenge if testing personnel were not adequately trained or did not have sufficient experience in performing syphilis rapid tests. Lack of appropriate lighting for interpreting test results and/or poor eyesight has also proven to result in false negative test interpretations. All rapid tests were read visually which made interpretation subjective, especially for weak reactive samples. A DPP micro reader was recently made available and the Chembio DPP test is intended to be used with the reader for objective result interpretation. False negative results could have a significant impact on patient management and risk of vertical (mother-to-child) and horizontal syphilis transmission. Technical support and training are critical to ensure the accuracy of rapid tests. BFP results are possibly associated with several patient characteristics (female, older age, pregnancy, intravenous drug use) and medical conditions including autoimmune disorders, cancers, malaria, other treponemal infections, HIV infection [31–34]. One BFP syphilis specimen was included for each EQA panel in this study and results were interpretated correctly at all POCT sites, showing no interference with the dual HIV/syphilis rapid tests at all POCT sites. Overall, DTS offered a reliable form of EQA for HIV and syphilis POC testing in remote settings in this study.

Our study has some limitations. DTS syphilis specimens were reported to produce expected results after storage at 2–8°C or at 18–24°C for up to 3 weeks and our panels likely fell within these temperature ranges during shipping to sites and storage [35]. However, DTS panels were not tested for reproducibility of results at different temperature and humidity levels in this study. Because of the study design limitation, no repeat testing of prepared panels, or follow-up with the participating site was possible to troubleshoot noted testing discrepancy. Turnover of staff at laboratories and, more specifically, at some clinical sites resulted in the need for ongoing supervision and training. The testing of panels occurred in different geographical areas including countries in Africa, Europe and South America and therefore, the results cannot be generalized to other regions. Dual HIV-syphilis rapid tests are used as the first test for pregnant women as part of the antenatal care but should be integrated into the overall algorithm for HIV testing for final diagnosis as recommended by WHO. This EQA study did not examine the quality of the overall HIV algorithm for pregnant women. However, this study, undertaken in association with a broad-based evaluation of POCTs, provides important information that could help authorities to design POCT evaluations in the future and the need to create a laboratory infrastructure capable of supporting the roll-out of a successful, quality POCT program through appropriate training, technical support, and technology transfer.

## Conclusions

Our external quality assessment to support the WHO ProSPeRo study for the evaluation of two dual HIV/syphilis point-of-care tests was performed in reference laboratories and their associated clinical sites in seven countries using HIV/syphilis serum liquid and DTS panels. Overall, laboratories demonstrated high EQA performance in this study. Both HIV/syphilis POCTs gave expected results in the clinic-based evaluations. DTS can be a useful and robust tool to monitor the quality of POC testing. However, the quality of testing varied at laboratories and sites suggesting the necessity for stringent quality assurance programs for POC testing. EQA results can be used to identify laboratories/sites where staff require continuous training.

## Data Availability

All data generated or analyzed during this study are included in this published article.

## Abbreviations

AIDS: Acquired immunodeficiency syndrome
BFP: Biological false positive
CDC: Centers for Disease Control and Prevention
CLIA: Clinical Laboratory Improvement Amendments
DPP: Dual Path Platform
DTS: Dry tube specimens
EMTCT: Elimination of mother-to-child transmission
EQA: External Quality Assessment
HIV: Human immunodeficiency virus
MSM: Men who have sex with men
MTCT: Mother-to-child transmission
PBS: Phosphate Buffered Saline
POCTs: Point-of-care Tests
PW: Pregnant women
ProSPeRo: Project on Sexually Transmitted Infection Point-of-Care Testing
RPR: Rapid Plasma Reagin
SHC: Syphilis Health Check
STDLRRB: STD Laboratory Reference and Research Branch
STIs: Sexually transmitted infections
*T. pallidum*: *Treponema pallidum subspecies* Pallidum
TP-HA: Treponema pallidum Haemagglutination Assay
TP-PA: *T. pallidum* Particle agglutination assay
WHO: World Health Organization

## References

[1] Global progress report on HIV, viral hepatitis and sexually transmitted infections, 2021 [https://www.who.int/publications/i/item/9789240027077]. Assessed 11 Jan 2022

[2] Sexually transmitted infections (STIs) [https://www.who.int/news-room/fact-sheets/detail/sexually-transmitted-infections-(stis)]. Assessed 11 Jan 2022.

[3] HIV/AIDS [https://www.who.int/news-room/fact-sheets/detail/hiv-aids]. Assessed Jan 2022.

[4] Wijesooriya NS, Rochat RW, Kamb ML, Turlapati P, Temmerman M, Broutet N, Newman LM (2016). Global burden of maternal and congenital syphilis in 2008 and 2012: a health systems modelling study. Lancet Glob Health.

[5] FACT SHEET – WORLD AIDS DAY 2023 [https://www.unaids.org/sites/default/files/media_asset/UNAIDS_FactSheet_en.pdf]. Assessed 1 Feb 2022.

[6] Elimination of mother-to-child transmission (EMTCT) of HIV and syphilis Global guidance on criteria and processes for validation [https://www.who.int/reproductivehealth/publications/rtis/9789241505888/en/]. Assessed 12 Jan 2022.

[7] Sexually Transmitted Disease Surveillance 2019 [https://www.cdc.gov/std/statistics/2019/default.htm]. Assessed 4 Mar 2022.

[8] HIV Surveillance Report 2019 [https://www.cdc.gov/hiv/pdf/library/reports/surveillance/cdc-hiv-surveillance-report-2018-updated-vol-32.pdf]. Assessed 4 Mar 2022.

[9] Syphilis and HIV: The Intersection of Two Epidemics [https://www.jwatch.org/ac201009030000001/2010/09/03/syphilis-and-hiv-intersection-two-epidemics]. Assessed 11 Jan 2022.

[10] Wu MY, Gong HZ, Hu KR, Zheng HY, Wan X, Li J (2021). Effect of syphilis infection on HIV acquisition: a systematic review and meta-analysis. Sex Transm Infect.

[11] Jarzebowski W, Caumes E, Dupin N, Farhi D, Lascaux AS, Piketty C, de Truchis P, Bouldouyre MA, Derradji O, Pacanowski J (2012). Effect of early syphilis infection on plasma viral load and CD4 cell count in human immunodeficiency virus-infected men: results from the FHDH-ANRS CO4 cohort. Arch Intern Med.

[12] Kofoed K, Gerstoft J, Mathiesen LR, Benfield T (2006). Syphilis and human immunodeficiency virus (HIV)-1 coinfection: influence on CD4 T-cell count, HIV-1 viral load, and treatment response. Sex Transm Dis.

[13] Lee MJ, Hallmark RJ, Frenkel LM, Del Priore G (1998). Maternal syphilis and vertical perinatal transmission of human immunodeficiency virus type-1 infection. Int J Gynaecol Obstet.

[14] Fleishman JA, Yehia BR, Moore RD, Gebo KA, Network HIVR (2010). The economic burden of late entry into medical care for patients with HIV infection. Med Care.

[15] Parekh BS, Ou CY, Fonjungo PN, Kalou MB, Rottinghaus E, Puren A, Alexander H, Hurlston Cox M, Nkengasong JN (2019). Diagnosis of Human Immunodeficiency Virus Infection. Clin Microbiol Rev.

[16] Dual HIV/syphilis rapid diagnostic tests can be used as the first test in antenatal care [https://www.who.int/publications/i/item/WHO-CDS-HIV-19.38]. Assessed 12 Jan 12, 2022.

[17] Gaitan-Duarte HG, Newman L, Laverty M, Habib NA, Gonzalez-Gordon LM, Angel-Muller E, Abella C, Barros EC, Rincon C, Caicedo S (2016). Comparative effectiveness of single and dual rapid diagnostic tests for syphilis and HIV in antenatal care services in Colombia. Rev Panam Salud Publica.

[18] Kasaro MP, Bosomprah S, Taylor MM, Sindano N, Phiri C, Tambatamba B, Malumo S, Freeman B, Chibwe B, Laverty M (2019). Field performance evaluation of dual rapid HIV and syphilis tests in three antenatal care clinics in Zambia. Int J STD AIDS.

[19] Toskin I, Blondeel K, Peeling RW, Deal C, Kiarie J (2017). Advancing point of care diagnostics for the control and prevention of STIs: the way forward. Sex Transm Infect.

[20] Network ProSPeRo (2020). Standardised protocol for a prospective cross-sectional multicentre clinic-based evaluation of two dual point-of-care tests for the screening of HIV and syphilis in men who have sex with men, sex workers and pregnant women. BMJ Open.

[21] Parekh BS, Anyanwu J, Patel H, Downer M, Kalou M, Gichimu C, Keipkerich BS, Clement N, Omondi M, Mayer O (2010). Dried tube specimens: a simple and cost-effective method for preparation of HIV proficiency testing panels and quality control materials for use in resource-limited settings. J Virol Methods.

[22] Hopkins AO, Trinh T, Fakile YF, Pillay A, Taylor MM, Kersh E, Kamb M (2020). Evaluation of the WHO/CDC Syphilis Serology Proficiency Programme to support the global elimination of mother-to-child transmission of syphilis: an observational cross-sectional study, 2008–2015. BMJ Open.

[23] Garner MF (1970). The biological false positive reaction to serological tests for syphilis. J Clin Pathol.

[24] R Core Team (2020). R: A language and environment for statistical computing. R Foundation for Statistical Computing, Vienna, Austria. [https://www.R-project.org/]. Assessed 3 Mar 2022.

[25] Rodriguez PJ, Roberts DA, Meisner J, Sharma M, Owiredu MN, Gomez B, Mello MB, Bobrik A, Vodianyk A, Storey A (2021). Cost-effectiveness of dual maternal HIV and syphilis testing strategies in high and low HIV prevalence countries: a modelling study. Lancet Glob Health.

[26] New reduced costs of dual HIV/syphilis rapid tests to accelerate progress toward elimination of mother-to-child transmission of HIV and syphilis [https://www.who.int/news/item/15-11-2021-new-reduced-costs-of-dual-hiv-syphilis-rapid-tests-to-accelerate-progress-toward-emtct-of-hiv-and-syphilis]. Assessed 12 Jan 2022

[27] Sexually Transmitted Infections Treatment Guidelines, 2021 [https://www.cdc.gov/std/treatment-guidelines/syphilis.htm]. Assessed 22 Jan 2022.

[28] Hamill MM, Mbazira KJ, Kiragga AN, Gaydos CA, Jett-Goheen M, Parkes-Ratanshi R, Manabe YC, Nakku-Joloba E, Rompalo A (2018). Challenges of rapid plasma reagin interpretation in syphilis screening in uganda: variability in nontreponemal results between different laboratories. Sex Transm Dis.

[29] Morshed MG, Singh AE (2015). Recent trends in the serologic diagnosis of syphilis. Clin Vaccine Immunol.

[30] Van Den Heuvel A, Smet H, Prat I, Sands A, Urassa W, Fransen K, Crucitti T (2019). Laboratory evaluation of four HIV/syphilis rapid diagnostic tests. BMC Infect Dis.

[31] Liu F, Liu LL, Guo XJ, Xi Y, Lin LR, Zhang HL, Huang SJ, Chen YY, Zhang YF, Zhang Q (2014). Characterization of the classical biological false-positive reaction in the serological test for syphilis in the modern era. Int Immunopharmacol.

[32] Nandwani R, Evans DT (1995). Are you sure it's syphilis? A review of false positive serology. Int J STD AIDS.

[33] Rompalo AM, Cannon RO, Quinn TC, Hook EW (1992). Association of biologic false-positive reactions for syphilis with human immunodeficiency virus infection. J Infect Dis.

[34] Arora S, Doda V, Rani S, Kotwal U (2015). Rapid plasma reagin test: high false positivity or important marker of high risk behavior. Asian J Transfus Sci.

[35] Benzaken AS, Bazzo ML, Galban E, Pinto IC, Nogueira CL, Golfetto L, Benzaken NS, Sollis KA, Mabey D, Peeling RW (2014). External quality assurance with dried tube specimens (DTS) for point-of-care syphilis and HIV tests: experience in an indigenous populations screening programme in the Brazilian Amazon. Sex Transm Infect.

